# Protocol for the PROSECCA study: a new approach for predicting radiotherapy outcome using artificial intelligence and electronic population-based healthcare data

**DOI:** 10.1136/bmjopen-2025-104408

**Published:** 2026-02-02

**Authors:** William H Nailon, David J Noble, Ewen Harrison, Zhuolin Yang, Sarah Elliot, Archie MacNair, George Beckett, Antony Hallam, Aziz Sheikh, Nicholas Mills, Roger Halliday, David Morrison, Anthony Chalmers, David Cameron, Charlie Gourley, Peter Hall, Christina Lilley, Linda J Carruthers, Michael Trainer, Donna Burns, Fiona Dee, Sankar Andiappa, Adrian Lonsdale, Megan Couper, Kirsty Farnan, John McLellan, Andrew Miller, Jacqueline Ogg, Jillian Moses, Steve Colligan, Graham MacDonald, Neil McPhail, Paddy Niblock, Nicholas MacLeod, Mike E Davies, Dave I Laurenson, James R Hopgood, Dorothy Boyle, Claire Paterson, Derek Grose, Iain Phillips, Stephen Harrow, Thomas Berger, Leila E A Shelley, Ian Sanders, Shelley Henderson, Aileen Duffton, Joanne Mitchell, Alasdair Rutherford, Duncan B McLaren

**Affiliations:** 1 Institute of Genetics and Cancer, University of Edinburgh Western General Hospital, Edinburgh, Lothian, UK; 2 Institute for Imaging, Data and Communications, The University of Edinburgh School of Engineering, Edinburgh, UK; 3 Oncology Physics, Edinburgh Cancer Centre, NHS Lothian, Edinburgh, UK; 4 Edinburgh Cancer Centre, NHS Lothian, Edinburgh, UK; 5 Usher Institute, University of Edinburgh Division of Clinical and Surgical Sciences, Edinburgh, UK; 6 UoE, Baynes Centre, Edinburgh Parallel Computing Centre, Edinburgh, UK; 7 Division of Community Health Sciences, University of Edinburgh Division of Clinical and Surgical Sciences, Edinburgh, UK; 8 Research Data Scotland, Baynes Centre, University of Edinburgh Institute of Governance, Edinburgh, UK; 9 Scottish Cancer Registry, Public Health Scotland, Edinburgh, UK; 10 Public Health Medicine, University of Glasgow, Glasgow, UK; 11 Institute of Cancer Sciences, University of Glasgow, Glasgow, UK; 12 Clinical Oncology, Beatson West of Scotland Cancer Centre, Glasgow, Glasgow, UK; 13 Medical Oncology, NHS Lothian, Edinburgh, UK; 14 Radiotherapy Physics, Newcastle Upon Tyne Hospitals NHS Foundation Trust Cancer Services, Newcastle upon Tyne, UK; 15 Radiotherapy Physics, NHS Tayside, Dundee, UK; 16 Radiotherapy, NHS Tayside, Dundee, UK; 17 Radiotherapy Physics, NHS Grampian, Aberdeen, UK; 18 Radiotherapy, NHS Grampian, Aberdeen, UK; 19 Radiotherapy Physics, NHS Highland, Inverness, UK; 20 Clinical Oncology, NHS Grampian, Aberdeen, UK; 21 Clinical Oncology, NHS Highland, Inverness, UK; 22 Clinical Oncology, NHS Tayside, Dundee, UK; 23 South East Scottish Cancer Research Network (SESCRN), NHS Lothian, Edinburgh, Edinburgh, UK; 24 Radiation Oncology, Centre Hospitalier Lyon Sud, Pierre-Bénite, Auvergne-Rhône-Alpes, France; 25 Medical Physics, NHS Tayside, Dundee, UK; 26 Beatson West of Scotland Cancer Centre, NHS Greater Glasgow and Clyde, Glasgow, UK

**Keywords:** Patients, RADIOTHERAPY, Prostate, Machine Learning, Urological tumours, Medical physics

## Abstract

**Introduction:**

Within the UK there are 33 deaths every day from prostate cancer, second only to lung cancer as the most common cause of cancer death in males in the UK. Of the 55 000 new cases each year, up to 50% of these patients will receive radiotherapy either alone or after prostatectomy. Although there have been significant improvements in the accuracy of radiotherapy delivery leading to better tumour targeting and a reduction in dose to normal tissues, significant permanent genito-urinary or gastrointestinal-related side effects are all too common. With nearly 80% of patients with prostate cancer surviving for 10 years or more, minimising life-limiting radiation damage to normal tissues is vitally important. However, at present, it is not possible to identify which patients will suffer a poorer outcome after radiotherapy. The aim of this study, improving radiotherapy in **PROS**tate cancer using **E**le**C**tronic population-based health**CA**re data (PROSECCA), is to do this by using the existing information in a patient’s digital healthcare record. By linking primary, secondary and tertiary clinical data, including digital image information, with radiotherapy treatment plans and outcome data, the PROSECCA study will identify de novo predictive biomarkers of radiation response and provide clinicians with a tool to individualise a radiotherapy dose and plan to maximise cure and minimise toxicity.

**Methods and analysis:**

The PROSECCA study is a large multidisciplinary project, the purpose of which is to analyse healthcare records from up to 15 000 patients with prostate cancer who underwent radiotherapy in the treatment of their cancer in Scotland between 2010 and 2022. Through the linkage of data obtained specifically for radiotherapy and data held within each patient’s unique electronic health record (EHR), the factors that indicate why some patients have a poor response to treatment, or an increased risk of side effects from radiation, will be identified. This will be made possible by the use of artificial intelligence and machine learning (AL/ML), which will help to identify at-risk patients earlier and allow adaptation of their treatment accordingly.

**Ethics and dissemination:**

The study is being conducted in accordance with the ethical principles set out in the Declaration of Helsinki and Good Clinical Practice that respects and protects the rights, and maintains confidentiality, of all trial participants. The study protocol (V.1.0) was reviewed by the South Central Oxford A Research Ethics Committee (REC) on 13 December 2021 and received a favourable opinion subject to each National Health Service (NHS) organisation confirming permission for patients treated within their area. Approval for the use of unconsented healthcare record data for patients included in the study and treated at one of the five Scottish Cancer Centres required an application to the NHS Scotland Public Benefit and Privacy Panel for Health and Social Care (HSC-PBPP). Full approval from the HSC-PBPP panel was received on 1 July 2024, which covered the use of pseudoanonymised EHR data for all patients participating in the study. The study is publicly listed on the NHS Health Research Authority site, with IRAS ID 306245 and REC reference 21/SC/0402. Dissemination of the study findings will take place through field-leading cancer, radiation oncology and medical physics journals. All manuscripts will be approved by the main study team and authorship determined by mutual agreement.

**Trial registration number:**

NCT06714630.

**STRENGTHS AND LIMITATIONS OF THIS STUDY**

- The PROSECCA study will include EHR data that are not currently taken into account in radiotherapy.
- The linkage of radiotherapy treatment planning data with mass digitalised healthcare records will create a range of longitudinal phenotype information for patients with prostate cancer treated in Scotland.
- All digitalised healthcare records held by Public Health Scotland (PHS) adhere to international coding standards accessible by a unique patient identifier.
- The cohort is currently restricted to patients treated in Scotland; however, plans are in place to establish performance on an independent cohort of patients treated across the UK.

## Introduction

 Between 2017 and 2019, prostate cancer accounted for 14% of all cancer deaths in the UK. This equates to over 12 000 men making it the second most common cause of cancer death in males.^1^ In the same period, over 55 000 men were diagnosed with prostate cancer, nearly half of whom received external beam radiotherapy as part of their treatment. In the majority of cases, radiotherapy is given as a curative treatment because of its effectiveness at destroying cancerous cells deep within the body. However, this comes at the cost of potentially damaging healthy, or normal, tissues close to the target.^1 2^

Current National Institute for Health and Care Excellence (NICE) guidelines recommend that prostate cancer is classified into three groups, low, intermediate and high, based on the risk of the disease spreading.^3^ In broad alignment with National Comprehensive Cancer Network (NCCN) guidelines, these groups are defined here as: low risk=prostate specific antigen (PSA)<10, Gleason Score≤6, T1-2a; intermediate risk=PSA 10–20, Gleason Score=7, or T2b-c; high risk=PSA>20, Gleason Score≥8, or T3-4.^4 5^ Tumour stage is based on digital rectal examination and the addition of either transrectal ultrasound, computerised tomographic (CT) and/or a magnetic resonance (MR) imaging. In Scotland, prostate biopsy is recommended for patients in the following categories: <50 years and PSA>3; 50–60 years and PSA>4; >60 years and PSA>5.

In managing patients with localised disease, the most common treatment options are radical prostatectomy and external beam radiotherapy, both of which are currently held at therapeutic equipoise.^6^,^9^ The focus of the PROSECCA study is on external beam radiotherapy where a linear accelerator has been used to deliver precise radiation doses to a predefined target volume over a number of days. Typically, doses in the range of 2–3 Gy are given each day, or fraction of treatment, over several weeks.^10^ More recently, hypofractionation treatments, which deliver much higher doses in fewer fractions, have been shown to be safe and effective for low and intermediate risk patients.^11^,^14^ However, irrespective of dose and fractionation schedule, life-limiting side effects from radiotherapy are common.^2 9^ The key to reducing side effects is to personalise an individual’s radiotherapy treatment. While there has been progress on doing this based on tumour biology as shown in the landmark study, validating predictive models and biomarkers of radiotherapy toxicity to reduce side-effects and improve quality-of-life in cancer survivors (REQUITE), such approaches, which require patients to provide biological samples, are expensive and not suitable for rolling out into everyday practice across the National Health Service (NHS).^15 16^ New approaches are required and this is the motivation for this study, improving radiotherapy in **PROS**tate cancer using **E**le**C**tronic population-based health**CA**re data (PROSECCA), which will use linked digital healthcare data acquired across a whole population to predict clinical outcomes for patients with prostate cancer undergoing radiotherapy. Currently, this is not done and little or no account is taken of the vast array of data that are available for each patient from their electronic health record (EHR), the extensive digital imaging data collected at diagnosis and during treatment, and during a course of radiotherapy.

The PROSECCA study aims to formalise an approach for identifying factors in a patient’s EHR that are reliable biomarkers of radiation-induced injury. In addition, the project will establish a centralised database containing radiotherapy data and linked healthcare records from patients with cancer treated in Scotland. In order to deliver on these goals, the project has a number of objectives:

### Nationwide radiotherapy-specific data and linked digital healthcare records

- The establishment of a nationwide database containing radiotherapy-specific data and linked healthcare records from patients with cancer treated in Scotland will serve as the test bed for the project. There is no such resource of this nature and scale currently available in the radiotherapy cancer community. The ability to link population-based differences in, for example, dose to tumour and normal tissue; fractionation schedules; treatment machines; cancer stage and grade; planning techniques to specific healthcare record information will be a significant outcome.

### Prediction of a patient’s risk of radiation toxicity and tumour control

- Using the linked dataset, predictive factors will be established indicating that a patient may have a poor response to treatment, including the increased risk of normal tissue toxicity. This information will be available both (1) prior to treatment and (2) during the course of treatment.
- Medium (5 year) and long (10 year) estimates of three clinically significant endpoints: (1) PSA relapse-free survival, (2) overall survival and (3) radiotherapy toxicity.
- To ensure maximum clinical impact, a software application, available to clinicians in the clinic, will be developed to use these predictive factors to identify (1) the most appropriate form of therapy, (2) the most appropriate dose and fractionation schedule and (3) the optimal time and how best to adapt treatment.
- To establish a pathway and methodology showing how the approach developed in PROSECCA can be applied to other tumour sites (eg, lung and head and neck cancer), thereby benefiting a much larger number of patients treated with radiotherapy.

## Methods and analysis

### Study design

**Figure 1:**
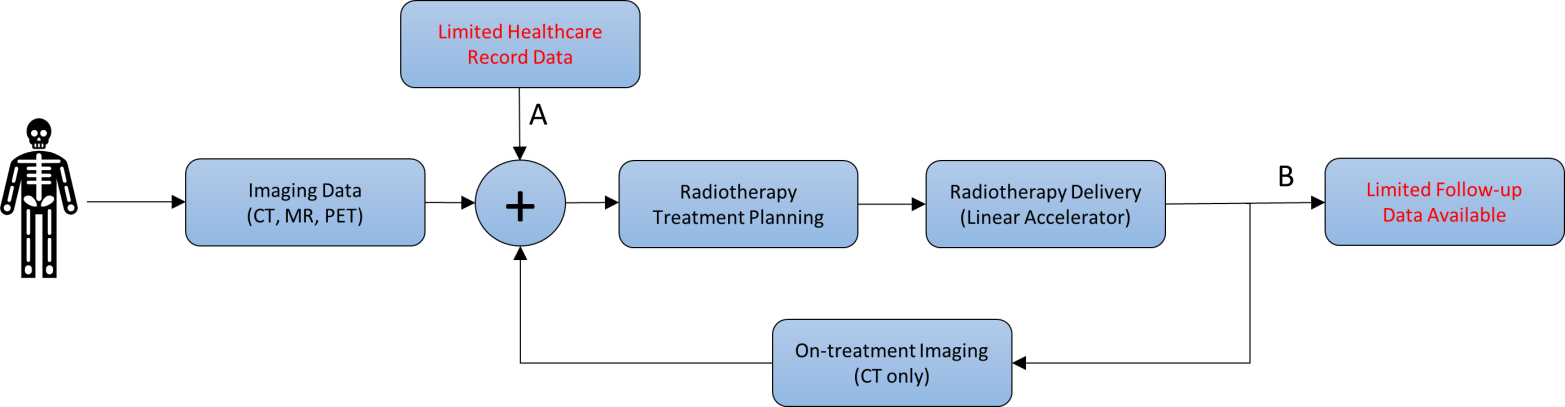
Graphical illustration of a typical radiotherapy workflow in which a patient undergoes imaging, typically CT, MRI and Positron Emission Tomography (PET), specifically for radiotherapy purposes. A: In general, creation of a radiotherapy plan uses a limited amount of information from a patient’s EHR. B: Additionally, little or no account of a patient’s EHR is considered at follow-up after treatment to establish what factors in a patient’s complex healthcare record indicate that they may have had a poor response to treatment, or an increased risk of side effects (toxicity) from radiation. With this knowledge, it would be possible to identify these patients earlier and adapt their treatment accordingly.

The PROSECCA study will improve prostate cancer treatment by utilising data within EHRs that is currently not used in preparation of radiotherapy treatment plans. This is possible because of the ability to link clinical intervention with International Classification of Diseases (ICD-10) codes and the relative ease with which mass longitudinal digitalised health records, including radiotherapy data, can be linked together in Scotland.^17^ The power of the Scottish digital healthcare infrastructure for answering population-based questions has been demonstrated in other large-scale health data research projects, although there has been limited work in cancer.^18 19^ Figures1  2 show the current approach used to plan and treat patients receiving radiotherapy and how the addition of information from a patient’s EHR will be used in the study.

**Figure 2:**
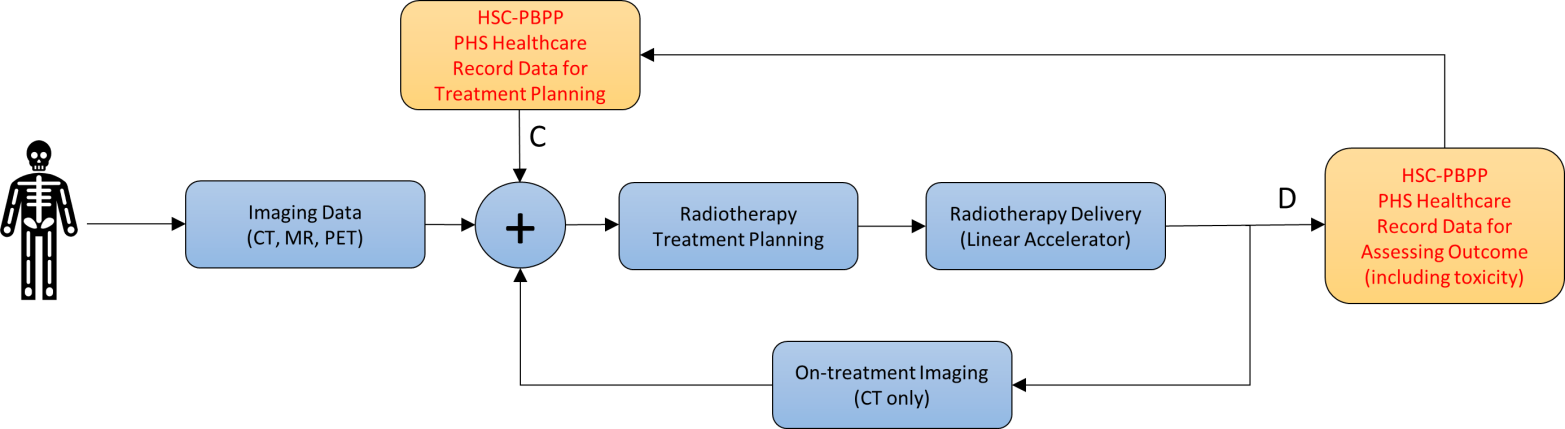
Graphical illustration of the radiotherapy workflow showing the inclusion of healthcare data from HSC-PBPP/PHS as proposed in the PROSECCA study. C: Here the creation of a radiotherapy plan uses significant personal information from a patient’s EHR. In addition, it will include information obtained from population-level analysis on the influence that other healthcare factors, which are documented in HSC-PBPP/PHS healthcare records, have on treatment response. D: Here, significant attention will be paid to a patient’s EHR after treatment to establish, and understand more about, the factors indicating that they may have had a poor response to treatment, or an increased risk of side effects (toxicity) from radiation. For example, it will be possible to study the protective effect that medications such as statins or antihypertensive medications have and how they may reduce radiotherapy-related toxicity.

**Figure 3:**
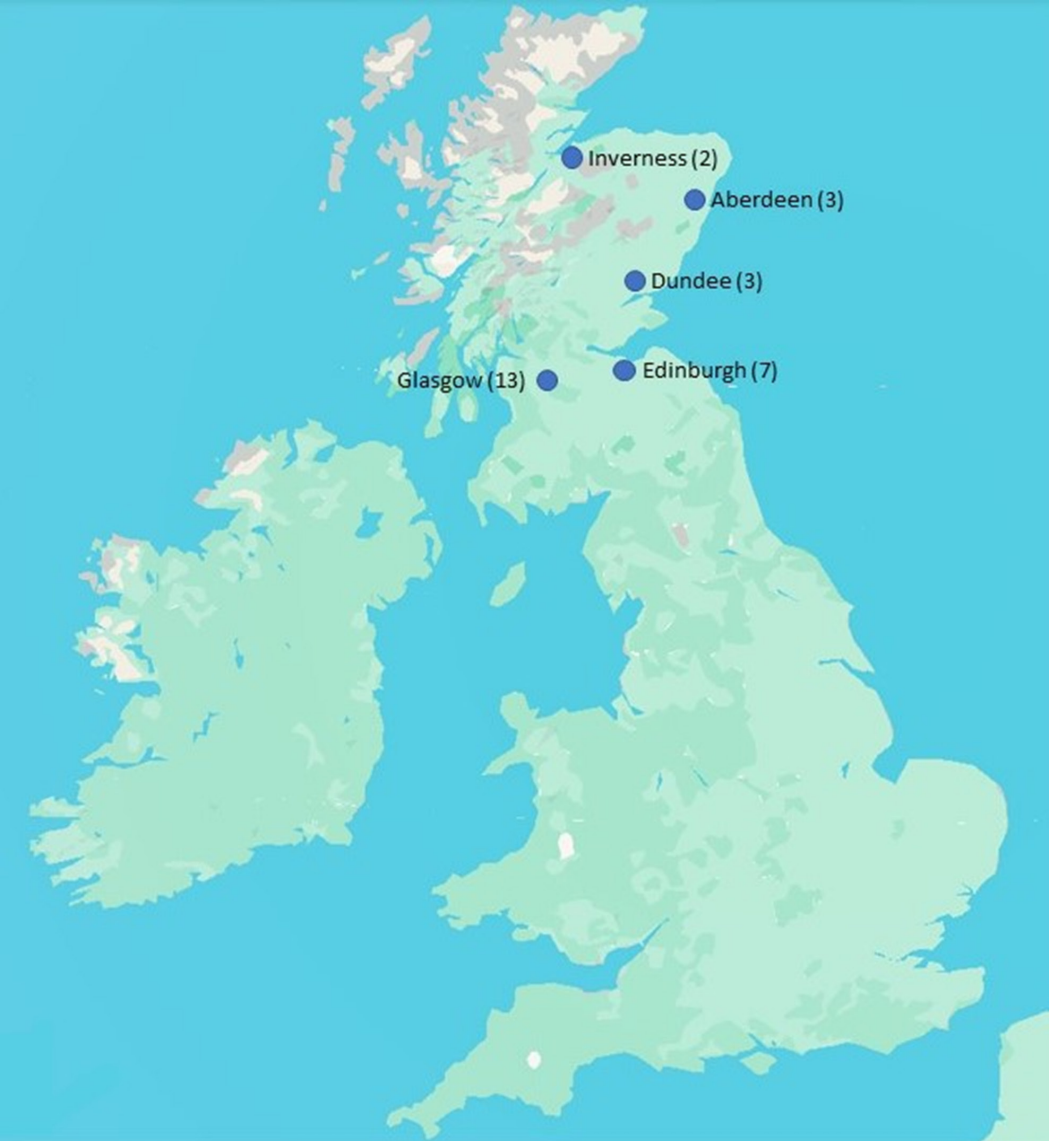
Map showing the location of the five Scottish Cancer Centres and in brackets the number of clinical linear accelerators at each location.

The study aims to include complete data sets from approximately 15 000 prostate patients who received radiotherapy at one of the five Scottish cancer centres, namely Aberdeen, Dundee, Edinburgh, Inverness and Glasgow (Figure 3). This estimate was based on an initial assessment of data from NHS Scotland Information Services Division (ISD), which indicated that over the proposed timeframe approximately 25 000 patients with prostate cancer were treated with radiotherapy in Scotland. It has been assumed that 40% of data will be incomplete or not suitable for the endpoints, Prostate Specific Antigen (PSA) relapse-free survival and overall survival, and that 60% of data will be incomplete or not suitable for the endpoint radiotherapy toxicity reported from all sources. From this, for each of the three key endpoints, the expected number of significant events in the study population was estimated to be: PSA relapse-free survival=1125; overall survival=2175; radiotherapy toxicity=1200. These preliminary estimates indicate that even with this level of incomplete data, there is still a sufficient number of complete data sets available to achieve the project goals. More details on how these figures were obtained are included in table 1.

**Table 1:** Preliminary estimates of the number of events for the three main endpoints of interest, PSA relapse-free survival, overall survival and radiotherapy toxicity as defined from all sources.

Endpoint of interest	Approach used to estimate events	Events
PSA relapse-free survival	5-year endpoint, 10% event rate based on the trial, a randomised phase III multi-centre trial of Conventional or Hypofractionated High dose Intensity modulated radiotherapy for Prostate cancer, (CHHiP) ^46^ Sample size of 11 250 based on 75% attrition in this data (75% of 15 000)	1125
Overall survival	10-year endpoint, 29% event rate based the trial, a Randomised controlled Trial of high dose versus standard dose conformal radiotherapy for localised prostate cancer, (RT01)^11 46 49^ Sample size of 7500 based on 50% attrition in this data (50% of 15 000)	2175
Radiotherapy toxicity (all sources)	5-year endpoint, 12% event rate for ≥ grade 2 based on CHIIP trial high grade group^11 46 49^ Sample size of 10 000 based on much higher attrition of 60% attrition for this endpoint of interest (60% of 25 000=10 000 followed by 12% of 10 000)	1200

Estimates were based on data from NHS ISD, Scotland, Prostate Cancer Incidence.

PSA, prostate specific antigen.

Each of the five Scottish cancer centres involved in the study uses the same commercial database for storing information on patients receiving radiotherapy for prostate cancer. This database, known as ARIA, is supplied by Varian Medical Systems^20^ and is used to maintain a complete independent record of a patient’s treatment, which is required by law under the Ionising Radiation (Medical Exposure) Regulations 2017 .^21^ Separately, Public Health Scotland (PHS), Scotland’s national public health body, holds health and social care data for the entire Scottish population (5.4 million) as far back as the 1970s. Since this time, every NHS patient treated in Scotland has been allocated a unique identifier at birth, or on registering with Scottish healthcare services. This 10-digit numeric code, known as the Community Health Index number (CHI), is the primary mechanism for patient identification for every healthcare episode that happens in Scotland, making it straightforward to access and find information about an individual patient.

**Figure 4:**
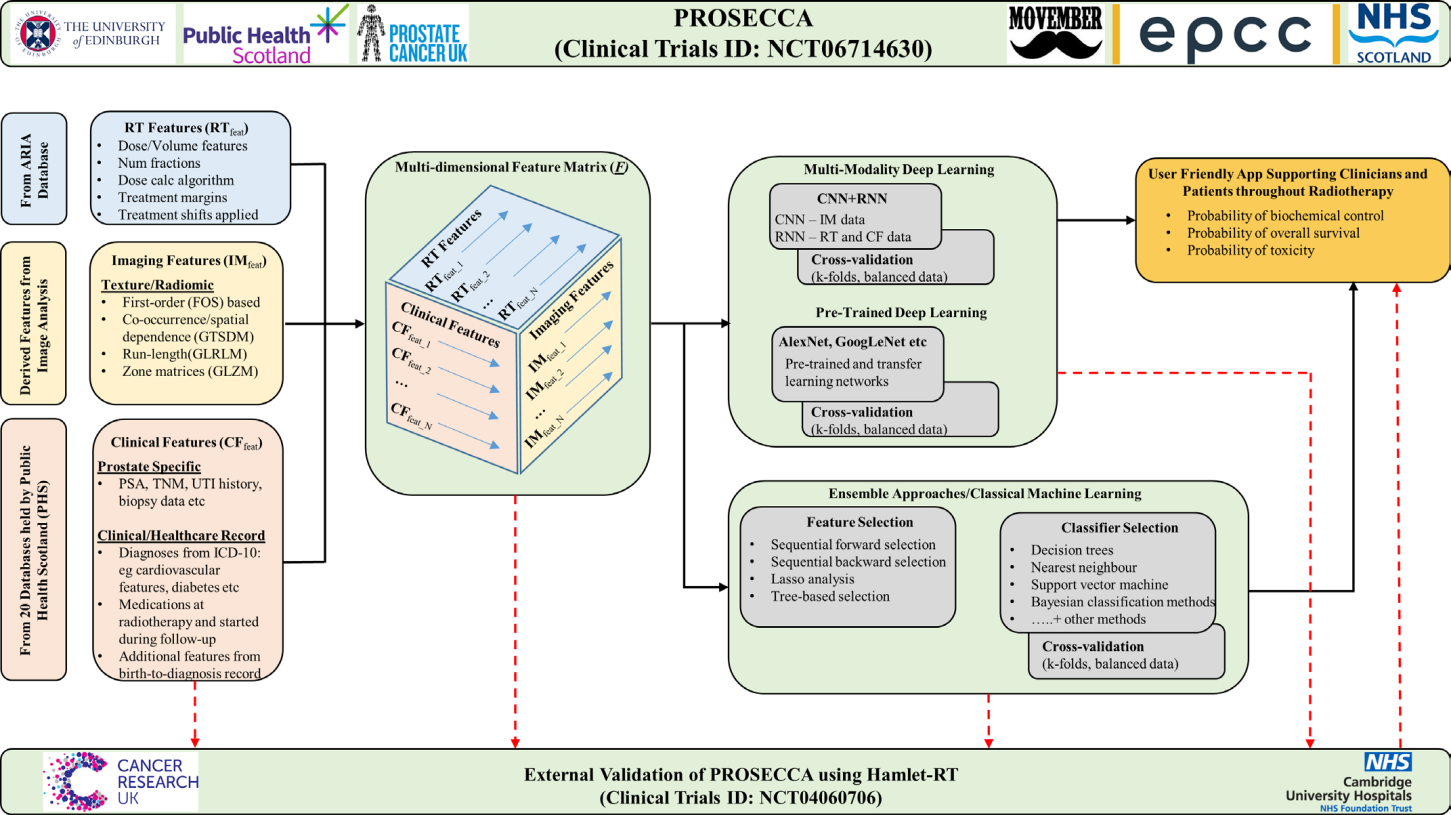
Detailed methodology of the PROSECCA study showing, from left to right, data entry into the platform from the ARIA radiotherapy information system and various healthcare data sets from PHS. All data processing will be carried out within the secure environment described in figure 5. CNN, Convolutional Neural Network; IM, Imaging; LASSO, least absolute shrinkage and selection operator; RNN, Recurrent Neural Network; TNM, Tumour, Node, Metastasis.

All healthcare records held by PHS are held within national datasets, which adhere to international standard coding definitions.^22^ Access to information held within a specific national dataset for research can be made through the Electronic Data Research and Innovation Service (eDRIS) team. The eDRIS team serves as the PHS point of entry for all projects, such as PROSECCA, that wish to use national datasets for research purposes. Central to the PROSECCA application to PHS was the identification of the national datasets of interest and the specific variables within these datasets that would be meaningful in assessing radiotherapy response. The list of national datasets held by PHS that will be accessed in the PROSECCA study, and linked to radiotherapy-specific information, is shown in table 2. Online supplemental table 4 also includes a brief justification for the use of each dataset, a requirement of the access request, and where available a recent scientific reference indicating the association with prostate cancer. Figure 4 illustrates how the data from the ARIA radiotherapy system and the data from the list of national datasets will be combined into a multidimensional array for further processing.

**Table 2:** Complete list of the national datasets held by PHS, and the number of variables within each, that will be accessed in the study.

National data set		Description	Number of unique features (n=431)
No.	Abbr. name		
1	SMR 00	Scottish Morbidity Record outpatient attendance	33
2	SMR 01	SMR general/acute inpatient and day case attendance	21
3	SMR 06	SMR Scottish Cancer Registry—The Scottish Cancer Registry and Intelligence Service has developed a national cancer intelligence platform that serves as a single point of entry to national cancer-specific data. The registry holds over 1 800 000 records dating back to 1958 when the registry began as well as data from the three cancer screening programmes in Scotland (bowel, breast and cervical)	107
4	GP Data	Coded GP-specific events that may be related to postradiation toxicity (eg, rectal haemorrhage, urinary infection and proctitis)	12
5	GP OOH	Coded GP out of hours—primary care out of hours data where specific events that may be related to post-radiation toxicity may be recorded (eg, rectal haemorrhage, urinary infection and proctitis)	24
6	NRS Deaths	National Records of Scotland deaths data	14
7	A&E2	Accident & Emergency	47
8	PIS	Prescribing Information Systems	16
9	SSCA	Scottish Stroke Audit	33
10	SBoSP	Scottish Bowel Screening Programme	25
11	SRR	Scottish Renal Registry	17
12	SHFA	Scottish Hip Fracture Audit	17
13	PDS	Dementia postdiagnostic support	10
14	DAISy	Drug and Alcohol Information System	12
15	SDMD, SMR25a, SMR25b	The Scottish Drug Misuse Database	11
16	WT	Waiting Times	7
17	SCS	Smoking Cessation Services	14
18	MS	Multiple Sclerosis	6
19	TRAK	Healthcare Information System	2
20	SPIDER	Streptococcus Pneumonia surveillance	3

### Study objectives

The primary objectives of the PROSECCA study are to use advanced artificial intelligence and machine learning (AI/ML) techniques to analyse the EHRs of patients with prostate cancer who underwent radiotherapy in the treatment of their cancer and to establish what factors indicate that a patient may have a poor response to treatment, or an increased risk of side effects from their treatment. The secondary objectives are to use these factors to build predictive models that identify these patients much earlier than is currently possible.

Figure 4 summarises the methodological approach of PROSECCA and the key parameters/features that will be used as input to different AI/ML models. With such an extensive dataset, the majority of which has not been analysed in any way, the preliminary phase of the study will use AI/ML frameworks already proven to work well in other big-data applications (eg, multimodal deep learning models, pretrained deep learning models and ensemble approaches). These will be adjusted to estimate biochemical relapse-free survival, overall survival and risk of toxicity from these data. Specifically, this will be approached by developing a number of ML models.

#### Multimodality deep learning models

Efficiently combining multimodality data within deep learning frameworks is challenging. The planned initial approach will involve building convolutional neural networks (CNNs) and vision transformers for imaging data and using other approaches for radiotherapy and clinical features, such as recurrent neural networks (RNNs).^23^ The output from these will be concatenated and passed to densely connected layers for classification. Labelled data will be available from: radiotherapy planning, medical imaging and EHR information (figure 4).

#### Pretrained deep learning models

It is likely that the performance of the imaging CNNs will be improved by using pretrained models and transfer learning models.^24^

#### Ensemble approaches/classical machine learning models

It is anticipated that the final classification will benefit from an ensemble approach using different AI/ML models to optimise classification. For example, decision trees, support vector machines, nearest neighbour and naive Bayes’ classifiers, all of which have been used extensively in previous radiotherapy and cancer classification problems.^25^,^31^

Serving as input data to these models will be unique information, or features, extracted from radiotherapy, imaging and EHR data. Currently, it is estimated that there will be, per time point of interest, more than 500 features of interest comprising:

- Radiotherapy planning features (n=20+ features).
  Including, but not limited to, dose to prostate and organs at risk, organ volume, number of fractions, delivery technique, dose calculation algorithm.
- Image analysis/radiomic features (n=150+ features).
  Including texture/radiomics analysis based on low-level and high-level feature analysis, features based on organ shape and position, features derived from other approaches such as Fourier analysis and wavelet analysis.
- Clinical/patient-specific features from EHRs (n=431 features at multiple timepoints).
  Including PSA, staging, Gleason score, age, smoking history, comorbidities including prescription medications via ICD-10 indicative of diseases such as cardiovascular and diabetes.

### Computational environment

**Figure 5:**
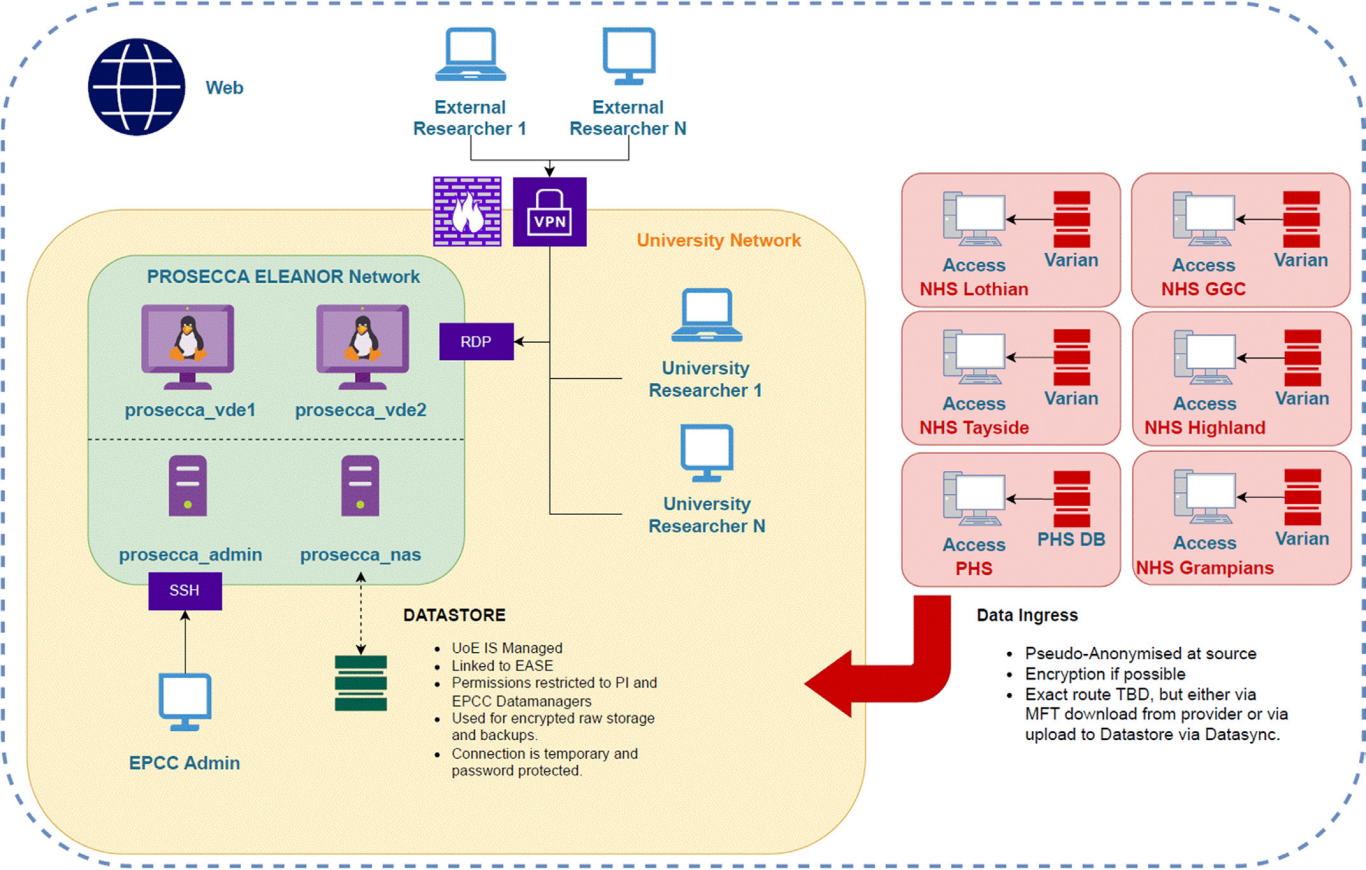
PROSECCA trusted research environment (TRE) showing connections from the Scottish Cancer Centres. Access is through a secure virtual private network (VPN), from where researchers can securely access the data and access a range of high-performance computing resources for rapid model development and testing. DB, database; EASE, University of Edinburgh's staff portal; GGC, Greater Glasgow and Clyde; MFT, multi-factor authentication; PI, principal investigator; RDS, Remote Desktop Connection; TBD, to be decided.

All data linkage and computation will be done within a secure virtual environment specially designed and built for the PROSECCA study by the Edinburgh Parallel Computing Centre (EPCC). This is one of the leading supercomputing centres in Europe hosting the UK’s Tier-1 research supercomputer as well as a number of other high-performance computing (HPC) systems including the Cirrus system, a national Tier-2 HPC service.^32^ The centre also hosts the first European deployment of a Cerebras CS-1 High Performance AI Computer, specifically designed to address the challenges of large-scale public health data sets. For the duration of the PROSECCA study, secure access to the data and to HPC computing resources will be available for researchers through this virtual environment. Figure 5 shows the key elements of this environment.

### External validation

A critically important step built in to the PROSECCA methodology is external validation. This will establish how well the model is calibrated and reacts to new unseen data and accounts for variability in the data collected from different patients, centres and at different time points. This has been taken into account by linking to Hamlet-RT, a prospective Cancer Research UK-supported study aimed at developing a way of predicting long-term side effects of radiotherapy (NCT04060706, REC 19/NE/0290).^33^ By deploying the algorithms developed on PROSECCA on the Hamlet-RT data set, it will be possible to validate performance on an independent UK-wide prostate cancer data set. In addition, all prediction models will be reported adhering to the consensus guidelines set out in Transparent Reporting of a multivariable prediction model for Individual Prognosis Or Diagnosis (TRIPOD)^34^ and as set out in the European Society for Radiotherapy and Oncology (ESTRO) position paper actively promoting this approach as part of the methodology required to bring clinical decision tools into the clinic.^35^

### Progress to date

To date, radiotherapy data from approximately 9500 patients has been collected and stored within the PROSECCA store and this will continue to grow over the coming months. However, one of the limiting factors of the study is that when radiotherapy data are removed from a clinical treatment planning system and transferred to a research environment, accessing key radiotherapy information is difficult without specialist computing skills. For example, information on the planned dose to an organ at risk from radiation, the volume of an organ receiving 95% of the total dose and the location of an organ on any day of treatment are all key parameters affecting patient response. While the current PROSECCA researchers have the necessary skills to interpret this data, it would be beneficial if a clinical treatment planning system was installed within the trusted research environment, which is planned for the future.

### Leadership and management

**Figure 6:**
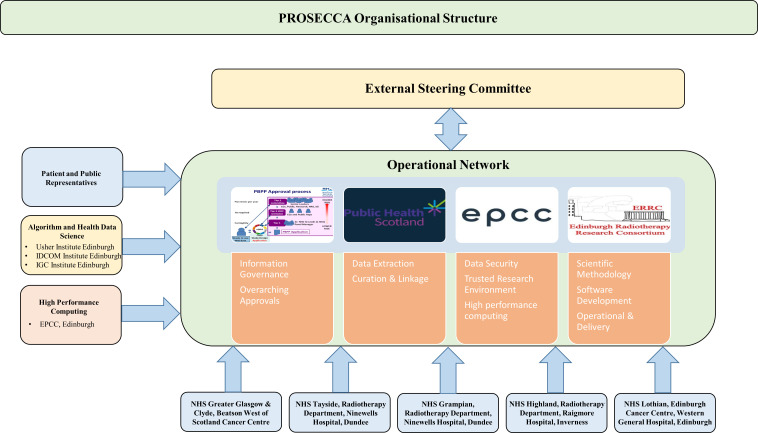
Organisational structure of the PROSECCA study showing the key groups contributing to the project. IGC, Institute of Genetics and Cancer.

The organisational and management structure of the project is shown in figure 6. Feeding into the main organisational network are representatives from the five Scottish cancer centres, EPCC, the Institute for Digital Imaging and Communication (IDCOM) at the University of Edinburgh and patient and public representatives. Oversight of all aspects of the project is provided by an independent steering committee made up of three experts with significant expertise in AI/ML in radiation oncology, clinical radiation oncology, healthcare data intelligence and prostate cancer. The experts are: Professor Raj Jena, Clinical Oncologist and Group Leader, Machine Learning & Radiomics in Radiation Oncology, University of Cambridge; Professor Yolande Lievens, Past-President of ESTRO, Chair of the Radiation Oncology Department at Ghent University Hospital and on the steering group of the E2-RADIATE project, an ESTRO initiative for research and big data collection; and Professor Sara Faithfull, Lead for Clinical Innovation and Expert in Health Sciences, particularly in prostate cancer, University of Surrey.^36^,^38^

To deliver the project, two research assistants (RA) have been employed for 3 years (ZY and SE), a further research assistant (TH) for 12 months spread over the duration of the project and a part-time administrator (ADMIN). ZY works specifically on AI/ML learning algorithm development for analysis of radiotherapy and digitalised healthcare data, and SE on data analysis techniques that can be used to identify primary endpoints for the AI/ML techniques developed by ZY. HPC support is provided by TH who is responsible for the development and maintenance of the secure computing environment used for the project (figure 5). Underpinning all aspects of PROSECCA is the ADMIN post required to maintain the smooth day-to-day running of the project. Online supplemental table 3 shows the meeting schedule and the groups invited to attend each meeting.

### Patient and public involvement

From the initial conceptualisation of the proposal, there has been patient and public involvement and engagement (PPIE). The original PPIE team offered guidance and codeveloped the submission by reviewing and providing feedback on early versions of the submission documentation. After funding was secured, two further members joined the group and have provided input through the steering committee and regular communication with the study team.

The PPIE team will continue to provide guidance and evaluate the study conduct on all levels. Their input will be particularly important as output from the study is prepared for presentation to lay audiences. One member of the PPIE team is also a past chair of a prostate support group and through this network will be able to disseminate information about the project. The team also intends to take advantage of the public engagement activities organised by Prostate Cancer UK (PCUK) to communicate the findings more widely.

### Protocol

#### Patient selection

Patients with prostate cancer who have received external beam radiotherapy with radical intent are eligible for inclusion in the PROSECCA study. Patients must have had PSA recorded at regular intervals after radiotherapy, a radiotherapy planning CT and corresponding radiotherapy and healthcare data available.

Patients who have received external beam radiotherapy as part of a palliative treatment schedule are not eligible for inclusion in the study, as well as those with no PSA information and follow-up healthcare and imaging data.

#### Consent

Access to unconsented health and social care data in Scotland is possible through a formal application to NHS Scotland Public Benefit and Privacy Panel for Health and Social Care (HSC-PBPP). Full permission to use electronic healthcare record data for all patients treated at the Scottish Cancer Centres participating in the study was granted by the HSC-PBPP panel on 1 July 2024. This is a national panel that considers the public benefit of every application and ensures that Information Governance (IG) standards align with the International Commissioner’s Office.^39^ Under article 6 (1)(e) of the General Data Protection Regulations (GDPR) the legal basis for the processing of the personal data used in the PROSECCA study is because it ‘*is necessary for the performance of the task carried out in the public interest*’.

#### Outcome measures

The primary outcome measures of the PROSECCA study are to establish what a patient’s probability of relapse-free survival, metastatic-free survival, cause-specific and overall survival will be when data from their EHR is combined with radiotherapy-specific parameters.

Secondary outcome measures include imaging-derived biomarkers and the independent and codependent predictive power of variables held within each patient’s EHR (online supplemental table 4) for prediction of primary outcomes.

Research and technical-scientific secondary outcomes include the curation of a population-based radiotherapy database containing imaging, dose and radiotherapy planning data and linked healthcare data from the national PHS archive. This also includes the establishment of a secure virtual environment, with high-performance computing capability, for rapid model development and outputs on the performance of a range of AI/ML models in predicting primary outcomes. Once established, this resource will also be used for future discovery research as well as validation of findings made by other researchers.

Patient-specific secondary outcomes will be in the form of a testbed for investigating the relationship between biochemical failure and radiotherapy-specific parameters such as dose, fractionation and planning techniques. This will also include how to personalise, or adapt, a patient’s course of radiotherapy by suggesting: the best form of therapy; the most appropriate dose and fractionation schedule; the optimal time to adapt; and the best mechanism for assessing any proposed personalised treatment. This will be available through an easy-to-use app.

## Discussion

The main aim of the PROSECCA study is to identify previously unrecognised patient-specific or treatment-specific factors that predict and improve outcome, reduce toxicity and maintain cure rates in men treated with radiotherapy for prostate cancer. With 80% of patients with cancer surviving for 10 years or more, it is important to ensure that these patients are not left with life-changing and/or life-limiting side effects from their radiotherapy. Moreover, long-term functional effects, including urinary incontinence, lower urinary tract symptoms and gastrointestinal symptoms, are vitally important to avoid in low-risk and intermediate-risk patients where 10-year mortality is less than 5%. However, limiting collateral damage to normal tissue without compromising the necessary high radiation doses to tumour volumes that ultimately cure patients is challenging. What is required is treatment personalisation, where an individual’s radiotherapy treatment is optimised specifically for them. To date, approaches to do this have been restricted to small numbers of carefully selected patients, are inordinately expensive and not suitable for everyday practice. The PROSECCA study proposes an alternative approach based on using existing information held within a patient’s EHR and the use of AI/ML to reveal new associations. By linking together radiotherapy data from 15 000+ patients with prostate cancer treated in Scotland, and information from 431 variables collected across 20 national data sets ±10 years from the onset of treatment, it will be possible to identify predictive factors that indicate when a patient may have a poor response to treatment, including the increased risk of normal tissue toxicity.

The ability to link primary, secondary and tertiary clinical data, including digital image information, with prostate radiotherapy treatment plans and outcome data (eg, PSA) will make it possible to identify population-based predictive biomarkers of treatment response. In preliminary work on a smaller representative dataset, the project team has reported that by adding AI-derived imaging biomarkers to the existing prediction models currently used in the NHS, improvements are possible.^40^ However, there are a number of underlying challenges, which are now discussed, that must be overcome before methods based on AI/ML can be moved from the research domain into the clinical domain.

There is a pressing need to develop, validate and translate reliable AI/ML models into all stages of the radiotherapy workflow. These approaches must be capable of handling different types of image data (eg, CT, MR and PET) and learning from new data/information. Despite the growing number of publications in this area, few make it beyond this stage because of the lack of robust external validation and institution-specific algorithm design. The PROSECCA study will contribute to this through the provision of data collected across multiple institutions, which will enable robust external validation and help establish the generalisability of the approaches. Furthermore, by deploying the methodology and AI/ML models developed in PROSECCA on the Hamlet-RT data, it will be possible to establish model robustness to new data.

The use of image analysis, or radiomics, in radiotherapy has grown because of the ability of these methods to serve as biological markers of tumour and normal tissue response to radiotherapy.^41^ However, adoption of these methods in the clinic to assess an individual’s susceptibility to radiation damage is not yet possible. Furthermore, many of the approaches widely in use are based on methods developed several decades ago.^42^ The scale of the radiotherapy imaging data available in PROSECCA will allow the power of new pixel-based image analysis approaches to be investigated for identifying underlying pathophysiology in images acquired before, during and after radiotherapy delivery.^40 43 44^

The evidence base on the use of large-scale cancer archives, or big data, for healthcare monitoring, quality improvement and decision making is emerging and recently researchers at The Institute for Cancer Research, London, used a big data approach to analyse the information from more than 700 patients treated with radiotherapy as part of the CHHIP trial. By linking medical history, genetics, radiotherapy dose and reported side effects, they investigated methods for treatment personalisation.^45 46^ In addition, there are active data collection programmes in prostate cancer such as the PIONEER project, a European network of excellence for big data in prostate cancer.^47^ While there is synergy between these initiatives and the PROSECCA study, the key differences are the scale and the relative ease with which mass longitudinal digitalised EHRs, including radiotherapy data, can be linked together in Scotland. As a result, where these previous studies have required years to collect very specific data, PROSECCA is amassing radiotherapy data from thousands of patients and linking these with information held by PHS in national datasets.

Accessing the data required for PROSECCA, which is generally stored in different databases and formats across organisations, is not only technically difficult but must align with the data protection laws on data minimisation as defined in Article 5 of the UK GDPR .^39^ Adherence to these standards is a requirement for access to health data in Scotland and through delegated authority from the NHS Scotland Chief Executive Officers and the Registrar General of National Records of Scotland these standards are upheld by HSC-PBPP. The purpose of the HSC-PBPP panel is to scrutinise requests for access to health data by researchers, organisations or individuals, and to act as the final arbiter in deciding whether it is appropriate for the request to be granted in full or in part. In this capacity, HSC-PBPP is not an ethical review body, and there is an expectation that requests have received favourable ethical reviews from an ethics panel before submission. While the panel works hard to strike a balance between safeguarding the privacy of those whose data are being released and making the best possible use of health and social care data for the benefit of the population, the sheer number of variables and datasets requested, combined with the complexity of the PROSECCA study, meant that this took a considerable amount of time. With initial discussions beginning in October 2022 and first access to linked health data in February 2025, all parties are aware that a more simplified and streamlined process for accessing public data would have societal and scientific benefits. Research Data Scotland (RDS), a not-for-profit charitable organisation, was set up in 2023 to address these issues and make it faster and simpler for researchers to access public sector data for research.^48^ The PROSECCA study has been used as a case study by the RDS team to demonstrate where this new approach to access may have been helpful.

The PROSECCA study aims to have a positive impact on the lives of men affected by prostate cancer, as well as a number of other areas including: the radiotherapy community, academic community, society, teaching and training. For those men affected by prostate cancer, the short-term impact of this work will be through the knowledge that there is a significant body of work going on that aims to obtain a deeper understanding of the role that all available healthcare data, including digital imaging information collected at diagnosis and treatment, has on improving outcome. The longer-term impact on these men will be through the creation of a clinical decision support software tool that will allow the impact on treatment from new parameters to be considered and allow additional decisions to be made on: the best form of therapy; the most appropriate dose and fractionation schedule based on biological data; the optimal time to adapt treatment (ie, before, during or after each fraction); and the most appropriate metrics for appraising any proposed personalised treatment.

## Supplementary material

10.1136/bmjopen-2025-104408 online supplemental table 1

10.1136/bmjopen-2025-104408 online supplemental table 2
