# Supplementary material for: Protocol for the PROSECCA study: a new approach for predicting radiotherapy outcome using artificial intelligence and electronic population-based healthcare data
Source: BMJ Open. 2026 Feb 2;16(2):e104408. doi: 10.1136/bmjopen-2025-104408 (PMC12878269; doi:10.1136/bmjopen-2025-104408)
Supplement: online supplemental table 1 [file bmjopen-16-2-s001.docx]

**Supplemental Table 3**

| Day-to-day informal meetings & communication | Usually in-person and email communication between the Principal Investigator, Research Assistants and Administrator. In addition, depending on the task, there will be Teams meetings between members of the core team. |
| --- | --- |
| Team Research Huddle | Weekly/Fortnightly team huddle involving the Principal Investigator, Research Assistants and Administrator. Other team members are invited as and when required. |
| Scientific Discussion | Monthly meetings at which all members of the Edinburgh team are invited to discuss/present on scientific matters relating to the project. |
| Progress Meeting | Monthly meeting at which all members of the Edinburgh team are invited to discuss operational matters and general progress relating to the project. |
| Heads of Radiotherapy Meeting | Quarterly meeting with the Heads of Radiotherapy from the five Scottish Cancer Centres to discuss radiotherapy-specific data issues as well as more general matters relating to the project. |
| Steering Committee Meeting | 6 monthly meeting at which the core team present to the PROSECCA external steering committee (Prof Raj Jena, Cambridge; Prof Yolande Leivens, Leuven, Prof Sara Faithful Guilford). PPI members also invited to attend in person. |
| Annual Meeting & Steering Committee Meeting | Annual meeting for the whole PROSECCA team. PPI members also invited to attend in person. |
