# Supplementary material for: Protocol for the PROSECCA study: a new approach for predicting radiotherapy outcome using artificial intelligence and electronic population-based healthcare data
Source: BMJ Open. 2026 Feb 2;16(2):e104408. doi: 10.1136/bmjopen-2025-104408 (PMC12878269; doi:10.1136/bmjopen-2025-104408)
Supplement: online supplemental table 2 [file bmjopen-16-2-s002.docx]

**Supplemental Material Table 4**

| **National Data Set** | | **Description** | **Number of Unique Features (N=431)** | **Justification** |
| --- | --- | --- | --- | --- |
| **Num** | **Abbr. Name** |  |  |  |
| 1 | SMR 00 | Scottish Morbidity Record outpatient attendance | 33 | A number of studies have investigated key risk factors in prostate cancer, for example, hypertension, BMI and socio-economic status. Access to SMR 00 and 01 before and after radiotherapy will allow the effect of these factors on radiotherapy to be fully investigated. |
| 2 | SMR 01 | SMR general/acute inpatient and day case attendance | 21 |  |
| 3 | SMR 06 | SMR Scottish Cancer Registry – The Scottish Cancer Registry and Intelligence Service (SCRIS) has developed a national cancer intelligence platform that serves as a single point of entry to national cancer-specific data. The registry holds over 1,800,000 records dating back to 1958 when the registry began as well as data from the three cancer screening programmes in Scotland (bowel, breast and cervical). | 107 | Access is required to ensure that there is complete information on the patient and the tumour (i.e., stage). |
| 4 | GP Data | Coded GP- specific events that may be related to post-radiation toxicity (e.g., rectal haemorrhage, urinary infection and proctitis) | 12 | In the absence of patient reported information to assess the efficacy of radiotherapy the PROSECCA study will identify radiation toxicity from healthcare records, rather than prospectively curated scoring systems often used in clinical trials. The project follows the published work of Lemanska and Faithful, to look for specific co-morbidities, medications and referrals, that correlate with radiation-related toxicity [1-3]. |
| 5 | GP OOH | Coded GP out of hours – primary care out of hours where specific events that may be related to post-radiation toxicity may be recorded (e.g., rectal haemorrhage, urinary infection and proctitis) | 24 | As in 4. |
| 6 | NRS Deaths | National Records of Scotland deaths data | 14 | For those patients who have died it is important to study the cause of death and to investigate this in the context of the radiotherapy treatment received. |
| 7 | A&E2 | Accident & Emergency | 47 | There will be patients presenting at A&E with conditions that are linked to prostate cancer either before or after radiotherapy. Access to A&E2 will ensure that patients presenting at A&E and being diagnosed with prostate cancer [4] and those arriving at A&E because of complications arising after radiotherapy [5] are identified. The most common radiation-induced complications for those presenting at A&E include haemorrhagic cystitis, urethral and ureteral strictures, urinary fistulae, and secondary primary malignancies. The PROSECCA study will provide a better understanding of the relationship between radiation dose and these complications [5]. |
| 8 | PIS | Prescribing Information Systems | 16 | There are a number of medications reported in the literature that have been noted as having a protective effect in reducing radiotherapy-related side-effects. For example, statins or antihypertensives [1-3]. By including information on these medications in PROSECCA it will be possible to quantify these effects. In addition, by including information on the specific timing, dose and frequency of medication more fine-grained analysis of these effects will be possible. |
| 9 | SSCA | Scottish Stroke Audit | 33 | An increased risk of stroke, transient ischemic attack (TIA), deep vein thrombosis (DVT) has been reported for men receiving treatment for prostate cancer [6]. There is also growing evidence that cancer and cardiovascular disease are connected through shared risk factors [7]. The inclusion this dataset will allow a comprehensive investigation of these associations to be carried out in the PROSSECCA cohort. |
| 10 | SBoSP | Scottish Bowel Screening Programme | 25 | There is evidence that long-term prostate cancer survivors who received radiotherapy as part of their treatment are at an increased risk of developing rectal cancer. Data from the SboSP will allow this to be investigated within the PROSECCA study [8]. |
| 11 | SRR | Scottish Renal Registry | 17 | Urological cancers such as prostate cancer have been reported as a potential risk in chronic kidney disease. Including data from the SRR will be an opportunity to investigate this within the Scottish population of prostate cancer patients undergoing radiotherapy in the treatment of their disease [9]. |
| 12 | SHFA | Scottish Hip Fracture Audit | 17 | Bone fractures are a common and problematic side-effect of pelvic (prostate) radiotherapy. Whilst there may be additional underlying mechanisms that affect this, for example osteoporosis, direct linkage of the SHFA dataset with prostate cancer patients treated within the PROSECCA study in Scotland has the potential to increase understanding in this area [10, 11]. |
| 13 | PDS | Dementia post-diagnostic support | 10 | There is an increased risk of dementia in patients receiving androgen deprivation therapy (ADT) in the treatment of prostate cancer [12]. ADT is used with radiotherapy to increase the curative potential of the treatment. Inclusion of data from the PDS dataset will allow an investigation of the correlations between dementia and prostate cancer treatment in patients within the PROSECCA study. |
| 14 | DAISy | Drug and Alcohol Information System | 12 | Prostate cancer survivors have been reported as having a significant increased risk of alcohol and drug use disorders leading to a suggestion that long-term psychological support, and detection of this condition, is required. Including data from this cohort will also allow specific properties of the radiotherapy treatment to be considered, which until now has not be taken into account as an influencing factor [13]. |
| 15 | SDMD, SMR25a, SMR25b | The Scottish Drug Misuse Database | 11 | As above in 14. |
| 16 | WT | Waiting Times | 7 | Inclusion of waiting time information will allow those patients not treated within the standard 62-day timeframe to be identified. |
| 17 | SCS | Smoking Cessation Services | 14 | Smoking has been suggested as having an inverse association with prostate cancer incidence, however, smokers have an increased risk of death from prostate cancer . To further understand this association in the Scottish prostate cancer cohort, the PROSECCA study will include information from the Scottish Cessation Services dataset [14]. |
| 18 | MS | Multiple Sclerosis | 6 | As recently reported in the literature the current association between MS and prostate cancer remains to be elucidated [15]. Inclusion of MS data from the MS audit dataset will allow the association between MS and the Scottish population of prostate cancer patients to be comprehensively investigated. |
| 19 | TRAK | Healthcare Information System | 2 | This is the healthcare information system for delivering, managing, and transforming care in Scotland. Access will be essential for clarifying data from patients included in the study. |
| 20 | SPIDER | Streptococcus Pneumonia surveillance | 3 | There is growing interest in the role of the intestinal microbiome in prostate cancer [16, 17]. Incorporating this information into the PROSECCA dataset will be a unique opportunity to investigate this linkage and to study its role with the Scottish prostate cancer sufferers |

**References**

1. Lemanska, A., et al., Linking CHHiP prostate cancer RCT with GP records: A study proposal to investigate the effect of co-morbidities and medications on long-term symptoms and radiotherapy-related toxicity. Tech Innov Patient Support Radiat Oncol, 2017. 2: p. 5-12.

2. Lemanska, A., et al., Symptom clusters for revising scale membership in the analysis of prostate cancer patient reported outcome measures: a secondary data analysis of the Medical Research Council RT01 trial (ISCRTN47772397). Quality of Life Research, 2017. 26(8): p. 2103-2116.

3. Lemanska, A., et al., Linkage of the CHHiP randomised controlled trial with primary care data: a study investigating ways of supplementing cancer trials and improving evidence-based practice. Bmc Medical Research Methodology, 2020. 20(1).52. McPhail, S., et al., Risk factors and prognostic implications of diagnosis of cancer within 30 days after an emergency hospital admission (emergency presentation): an International Cancer Benchmarking Partnership (ICBP) population-based study. Lancet Oncology, 2022. 23(5): p. 587-600.

4. McPhail, S., et al., Risk factors and prognostic implications of diagnosis of cancer within 30 days after an emergency hospital admission (emergency presentation): an International Cancer Benchmarking Partnership (ICBP) population-based study. Lancet Oncology, 2022. 23(5): p. 587-600.

5. Chorbinska, J., et al., Urological complications after radiation therapy-nothing ventured, nothing gained: a Narrative Review. Translational Cancer Research, 2021. 10(2): p. 1096-1118.

6. Deka, R., et al., Stroke and Thromboembolic Events in Men with Prostate Cancer Treated with Definitive Radiation Therapy with or without Androgen Deprivation Therapy. International Journal of Radiation Oncology Biology Physics, 2019. 105(1): p. E273-E274.

7. Willems, R.A.L., et al., Evolving data on cardiovascular complications in cancer. Thrombosis Research, 2022. 213: p. S87-S94.

8. Omer, D.M., et al., Rectal Cancer after Prostate Radiation: A Complex and Controversial Disease. Cancers, 2023. 15(8).

9. Ciorcan, M., et al., The Impact of Chronic Kidney Disease on the Mortality Rates of Patients with Urological Cancers-An Analysis of a Uro-Oncology Database from Eastern Europe. Journal of Personalized Medicine, 2023. 13(11).

10. Elliott, S.P., et al., Three-dimensional external beam radiotherapy for prostate cancer increases the risk of hip fracture. Cancer, 2011. 117(19): p. 4557-65.

11. Chatzimavridou-Grigoriadou, V., et al., Radiotherapy-related insufficiency fractures and bone mineral density: what is the connection? Endocr Connect, 2023. 12(7).

12. Nead, K.T., S. et al., Androgen deprivation therapy for prostate cancer and dementia risk: a systematic review and meta-analysis. Prostate Cancer Prostatic Dis, 2017. 20(3): p. 259-264.

13. Crump, C., et al., Risks of alcohol and drug use disorders in prostate cancer survivors: a national cohort study. JNCI Cancer Spectr, 2023. 7(4).

14. Al-Fayez, S, et al., Cigarette smoking and prostate cancer: A systematic review and meta-analysis of prospective cohort studies. Tob Induc Dis, 2023. 21: p. 19.

15. Hu, Z.Y., et al., Association between multiple sclerosis and prostate cancer risk: A systematic review and meta-analysis. Oncology Letters, 2023. 25(2).

16. Fujita, K., et al., Gut microbiome and prostate cancer. International Journal of Urology, 2022. 29(8): p. 793-798.

17. Fujita, K., et al., The role of gut microbiome in prostate cancer. Cancer Science, 2023. 114: p. 1542-1542.
