# Supplementary material for: Protocol for the PROSECCA study: a new approach for predicting radiotherapy outcome using artificial intelligence and electronic population-based healthcare data
Source: BMJ Open. 2026 Feb 2;16(2):e104408. doi: 10.1136/bmjopen-2025-104408 (PMC12878269; doi:10.1136/bmjopen-2025-104408)
Supplement: Supplementary file 3 [file bmjopen-2025-104408.reviewer_comments.pdf]

# PEER REVIEW HISTORY

BMJ Open publishes all reviews undertaken for accepted manuscripts. Reviewers are asked to complete a checklist review form and are provided with free text boxes to elaborate on their assessment. These free text comments are reproduced below.

## ARTICLE DETAILS

### Title (Provisional)

Protocol for the PROSECCA study: a new approach for predicting radiotherapy outcome using artificial intelligence and electronic population-based healthcare data

### Authors

Nailon, B Henry; Noble, David J; Harrison, Ewen; Yang, Zhuolin; Elliot, Sarah; MacNair, Archie; Beckett, George; Hallam, Antony; Sheikh, Aziz; Mills, Nicholas; Halliday, Roger; Morrison, David; Chalmers, Anthony; Cameron, David; Gourley, Charlie; Hall, Peter; Lilley, Christina; Carruthers, Linda J; Trainer, Michael; Burns, Donna; Dee, Fiona; Andiappa, Sankar; Lonsdale, Adrian; Couper, Megan; Farnan, Kirsty; McLellan, John; Miller, Andrew; Ogg, Jacqueline; Moses, Jillian; Colligan, Steve; MacDonald, Graham; McPhail, Neil; Niblock, Paddy; MacLeod , Nicholas; Davies, Mike E; Laurenson, Dave I; Hopgood, James R; Boyle, Dorothy; Paterson, Claire; Grose, Derek; phillips, iain; Harrow, Stephen; Berger, Thomas; Shelley, Leila EA; Sanders, Ian; Henderson, Shelley; Duffton, Aileen; Mitchell, Joanne; Rutherford, Alasdair; McLaren, Duncan B

---

## VERSION 1 - REVIEW

---

| | |
|---|---|
| **Reviewer** | **1** |
| **Name** | **Cowan, Janet E** |
| **Affiliation** | **University of California San Francisco, Urology** |
| **Date** | **12-Jun-2025** |
| **COI** | **None** |

---

Manuscript id bmjopen-2025-104408

This paper reports on the PROSECCA study of prostate cancer patients treated with radiotherapy from 2010 to 2022 at 5 cancer centers in Scotland. Objectives: (1) create a data repository for existing clinical EHR, radiotherapy delivery, and digitized imaging for up to 15,000 patients; (2) provide a platform for AIML analysis of the repository data set. The goal is for investigators to use this resource to develop biomarkers to identify men at risk for poorer outcomes (biochemical, treatment-related toxicity, survival) after radiotherapy.

The authors describe an impressive study. My concerns are about the quality and lack of follow data which would be necessary to develop biomarkers and decision support tools. The report states:

"It has been assumed that 40% of data will be incomplete or not suitable for the endpoints, PSA relapse free survival and overall survival, and that 60% of data will be incomplete or not suitable for the endpoint radiotherapy toxicity reported from all sources."

• How will investigators achieve the stated goals if half of the outcomes data is deemed usable/complete?

• Please discuss why values/follow up are missing. Presumably a large group of patients would be censored before the outcome event.

• Describe efforts to improve completeness of outcomes data, information that is essential for this report.

• Report duration of post-radiation follow up for the 9,500 participants to date

General comment

Please correct typo in the sentence with footnote 42.

---

## VERSION 1 - AUTHOR RESPONSE

**Reviewer 1: Comments to the Author**

We are very grateful for the helpful and constructive comments from Reviewer 1 and for the opportunity to significantly improve our manuscript based on these comments. The following information is provided in response to each of the points raised. Any new text that appears in the manuscript is highlighted in red.

*The authors describe an impressive study. My concerns are about the quality and lack of follow data which would be necessary to develop biomarkers and decision support tools. The report states:*

*"It has been assumed that 40% of data will be incomplete or not suitable for the endpoints, PSA relapse free survival and overall survival, and that 60% of data will be incomplete or not suitable for the endpoint radiotherapy toxicity reported from all sources."*

*How will investigators achieve the stated goals if half of the outcomes data is deemed usable/complete?*

Thank you for raising this point and my apologies on behalf of the whole team for the lack of clarity that presentation of the endpoint data in this way has caused. The PROSECCA proposal has matured over a number of years and over this time has benefitted greatly from discussions with a large number of professionals in the field. One of the recurring themes of these exchanges has been the quality of the data, in particular managing incomplete data, exactly as raised here by

Reviewer 1. Specifically, the question raised from our discussions has been "*How we can guarantee the quality of the data that we include in our models to ensure that this is truly representative for the scientific questions being asked*?". This is indeed broadly similar to the points raised by Reviewer 1 and the description below describes the origins of the outcome data estimates and also the reasons why this information was included in the first instance, which we now realise is confusing without further clarifications.

To take account of incomplete data at the time of study design, and also at the time of the original grant submission, we had to make generous over-estimates as to the potential impact of incomplete/missing data to ensure that we would still have sufficient power within our study. Furthermore, in generating these estimates it was also important to use quantifiable examples taken from measures that are well known to be associated with clinical outcomes. To do this we analysed data from NHS Scotland Information Services Division (ISD) over the proposed timeframe of the study. Using ISD data from 25,000 patients treated by radical radiotherapy over the time frame of the study, we estimated that incomplete toxicity data would be found in approximately 60% patients leaving a potential figure of 10,000 patients to analyse within our population. Similarly, we estimated that 40% of patients may not have sufficient PSA follow up data or correctly attributable cause specific survival data leaving 15,000 patients to analyse within our population. From these population estimates, a calculation of potential clinically relevant events was made based on outcomes from a published clinical trial data (see Table 1 of the main manuscript). At 5 years, the number of patients predicted to have failed their radiotherapy was estimated to be 10% (1500 out of a 15,000 population). Assuming the worst-case scenario of 25% inadequate data leaves a minimum of 1125 biochemically relapsed patients to analyse. Similarly for overall and cause specific survival at 10 years we estimated that 29% patients may have died. The exact cause of death may not have been correctly attributed to prostate cancer in a generous 50% of cases leaving 7500 patients to analyse and 2175 events. Lastly 5-year grade 2 or higher radiotherapy toxicity was estimated to be 12% of patients. A worst-case scenario of 60% lack of data would leave 10,000 patients to analyse with an estimated 1200 toxicity events. This preliminary analysis allowed us to estimate that there would be more than 1000 patients/cases in each group and ensure that there was sufficient power within our study. The groups and number of patients within each group were estimated to be:

- PSA relapse free survival (PSA) = 1125 patients;
- Overall survival (OS) = 2175 patients;
- Radiotherapy toxicity (RADtox)= 1200 patients.

Whilst Reviewer 1 asks, quite rightly, if it will be possible to achieve the stated goals of the study when only half of the outcome data is deemed usable/complete the main reason for including this information in the first instance was to highlight that even with the loss of more than half of the patients with complete outcome data, the total number of patients/cases is still highly significant with more than 1000 patients in each category (PSA=1125, OS=2175, RADtox=1200). Furthermore, it is commonly reported that AI or deep learning approaches require large numbers of cases for training and testing. Once again, the rationale for reporting these estimates was to help reviewers appreciate that the PROSECCA study, with aim of including over 15,000 prostate patient data sets, was appropriately powered for the questions being asked.

In order to clarify that even with these significant data losses there is still sufficient data to achieve the project goals, the following text has been added to the Study Design section

**Study Design (Original Text)**

The study aims to include complete data sets from approximately 15,000 prostate patients who received radiotherapy at one of the five Scottish cancer centres, namely Aberdeen, Dundee, Edinburgh, Inverness and Glasgow (Figure 3). This estimate was based on an initial assessment of data from NHS Scotland Information Services Division (ISD), which indicated that over the proposed timeframe approximately 25,000 prostate cancer patients were treated with radiotherapy in Scotland. It has been assumed that 40% of data will be incomplete or not suitable for the endpoints, PSA relapse free survival and overall survival, and that 60% of data will be incomplete or not suitable for the endpoint radiotherapy toxicity reported from all sources. From this, for each of the three key endpoints, the expected number of significant events in the study population was estimated to be: PSA relapse free survival = 1125; overall survival = 2175; radiotherapy toxicity = 1200. More details on how these figures were obtained are included in Table 1.

**Study Design (Revised Text)**

The study aims to include complete data sets from approximately 15,000 prostate patients who received radiotherapy at one of the five Scottish cancer centres, namely Aberdeen, Dundee, Edinburgh, Inverness and Glasgow (Figure 3). This estimate was based on an initial assessment of data from NHS Scotland Information Services Division (ISD), which indicated that over the proposed timeframe approximately 25,000 prostate cancer patients were treated with radiotherapy in Scotland. It has been assumed that 40% of data will be incomplete or not suitable for the endpoints, PSA relapse free survival and overall survival, and that 60% of data will be incomplete or not suitable for the endpoint radiotherapy toxicity reported from all sources. From this, for each of the three key endpoints, the expected number of significant events in the study population was estimated to be: PSA relapse free survival = 1125; overall survival = 2175; radiotherapy toxicity = 1200. These preliminary estimates indicate that even with this level of incomplete data, there is still sufficient numbers of complete data sets available to achieve the project goals. More details on how these figures were obtained are included in Table 1.

- Please discuss why values/follow up are missing. Presumably a large group of patients would be censored before the outcome event.

- Describe efforts to improve completeness of outcomes data, information that is essential for this report.

Thank you for raising these important points, which we have grouped together for the purposes of our response. As mentioned in the manuscript, the PROSECCA project aims to analyse complete healthcare records from up to 15,000 prostate cancer patients who underwent radiotherapy in the treatment of their cancer in Scotland between 2010 and 2022. This involves linking each patient's radiotherapy record with their digital healthcare record. In general follow-up for prostate cancer patients treated in Scotland involves attendance at an outpatient clinic at typically 3, 6, 12, 24, 36, 48 and 60 months after treatment where standard clinical outcome data is acquired. These appointments would have been a combination of hospital clinic attendance with the cancer team and subsequent community GP follow up. As a result, there is no routine collection of radiotherapy-specific follow-up information at these clinics, for example, CTCAE/RTOG (Common Terminology Criteria for Adverse Events/Radiation Therapy Oncology Group) scores for classification and grading of (radiation) side effects. This addresses Reviewer 1's point about why values/follow up are missing. To address this issue PROSECCA uses a new approach of inferring clinical toxicity by data/events recorded within a patient's digital healthcare record and coded by Public Health Scotland. The fact that we can do this at all is due to the unique CHI number and excellent digital informatics infrastructure within Scotland. An example of inferred toxicity would be patients presenting at an Accident and Emergency (A&E) department because of complications arising after radiotherapy (e.g., haemorrhagic cystitis, urethral and ureteral strictures, urinary fistulae, and secondary primary malignancies) where they will have all aspects of their admission digitally coded. It will then be possible to identify these patients in the study and link planned radiation dose at the voxel level with a specific clinical outcome (e.g., a urethral complication). Similarly, there is evidence that long-term prostate cancer survivors who received radiotherapy as part of their treatment are at an increased risk of developing rectal cancer. Therefore, data from the Scottish Bowel Screening Program (SboSP) will allow this to be investigated within the PROSECCA study and assess quantitatively the impact that radiotherapy has on the long-term risk of bowel cancer. In addition, by having access to the digital Prescribing Information System (PIS) data for NHS Scotland, it will be possible to identify patients prescribed medications that have been noted as having a protective effect in reducing radiotherapy-related side-effects, for example statins or antihypertensives. By including information on medications in PROSECCA it will be possible to quantify these effects.

An important aspect of addressing the completeness of the data is the sheer number of digitally coded healthcare variables that are held across 20 different data sets by PHS. These variables have multiple entries spread across the time period of PROSECCA leading to a data file of several million entries. Table 2 in the original manuscript lists the national datasets held by Public Health Scotland that will be accessed in the study. Also included is the number of variables within each dataset and a brief justification for the access request and where appropriate a recent scientific reference indicating the association with prostate cancer. This addresses our response to Reviewer 1's point, Describe efforts to improve completeness of outcomes data, information that is essential for this report.

Since the original submission of the manuscript, we have taken receipt of our first batch of data from Public Health Scotland. Whilst we are still curating and absorbing the contents of this data it has allowed us to begin building a picture of the diverse range of radiation toxicities that we will be able to investigate with data on this scale. This also adds to our response to Reviewer 1's first point, about how the stated goals will be achieved. Our initial assessment is that we have sufficient data to go beyond the original toxicity endpoints listed above because we have more fine-grained information on a number of specific radiation toxicities, which are shown in the table below.

| **Toxicity Name** | **Inferred toxicity at 3 m** | **Inferred toxicity at 6 m** | **Inferred toxicity at 12 m** |
|---|---|---|---|
| Constipation | 71 | 115 | 209 |
| Cystitis | 54 | 93 | 158 |
| Diarrhoea | 8 | 13 | 31 |
| Fracture | NA | NA | 2 |
| Haematuria | 10 | 35 | 68 |
| Haemorrhage | 13 | 19 | 77 |
| Haemorrhoids | 7 | 11 | 34 |
| Proctitis | 17 | 36 | 187 |
| Prostatitis | 14 | 26 | 30 |
| Ulcer | 1 | 3 | 8 |
| Urinary Tract Infection | 60 | 103 | 198 |

**Table**: Preliminary analysis of the PROSECCA digital healthcare file showing the number of patients with a range of toxicities recorded at 3, 6 and 12 months after radiotherapy.

Whilst it would have been useful to report this range of toxicities in the original grant proposal, it simply wasn't possible to obtain this data and establish how many events would be available for each end point of interest. This is another reason for using PSA, OS, and RADtox in our initial estimates to simply demonstrate the scale of the data available. Over the next few months the team will continue mining the data to build robust estimates of the number of events at each endpoint of interest and for a range of timepoints.

• Report duration of post-radiation follow up for the 9,500 participants to date

As indicated in our responses above post-radiation follow-up data will be available until the point of data extraction by Public Health Scotland. With future data extractions by Public Health Scotland planned until 2027 this will mean that there will be at the very least 5 years of follow-up data for the participants. However, for a very large proportion of patients there will be 10 years of follow-up and in many cases more.

General comment

Please correct typo in the sentence

with footnote 42. Thank you for highlighting this, it has now been addressed.

Thank you once again for considering our article and for giving us the opportunity to respond to these very helpful comments and to improve our manuscript.

If you require any further information, please do not hesitate to contact me With kind regards.

## VERSION 2 - REVIEW

| | |
|---|---|
| **Reviewer** | **1** |
| **Name** | **Cowan, Janet E** |
| **Affiliation** | **University of California San Francisco, Urology** |
| **Date** | **24-Sep-2025** |
| **COI** | |

Thank you for addressing each of my comments. I recommend this version for publication.
